# Aptamer-guided extracellular vesicle theranostics in oncology

**DOI:** 10.7150/thno.39706

**Published:** 2020-02-21

**Authors:** Phuong H-L Tran, Dongxi Xiang, Tuong N-G Nguyen, Thao T-D Tran, Qian Chen, Wang Yin, Yumei Zhang, Lingxue Kong, Andrew Duan, Kuisheng Chen, Miomio Sun, Yong Li, Yingchun Hou, Yimin Zhu, Yongchao Ma, Guoqin Jiang, Wei Duan

**Affiliations:** 1 School of Medicine and Centre for Molecular and Medical Research, Deakin University, Waurn Ponds, Victoria, Australia; 2 Division of Genetics, Department of Medicine, Brigham and Women's Hospital/Harvard Medical School, 77 Avenue Louise Pasteur, Boston, MA 02115, USA; 3 Department for Management of Science and Technology Development, Ton Duc Thang University, Ho Chi Minh City, Vietnam; 4 Faculty of Pharmacy, Ton Duc Thang University, Ho Chi Minh City, Vietnam; 5 Translational Medical Center, The Chinese People's Liberation Army General Hospital, 28 Fuxing Road, Haidian District, Beijing, China, 100853; 6 Institute for Frontier Materials, Deakin University, Waurn Ponds, Victoria, 3216, Australia; 7 School of Medicine, Faculty of Medicine, Nursing and Health Sciences, Monash University, 27 Rainforest Walk, Clayton VIC 3800, Australia; 8 Department of Pathology, The First Affiliated Hospital, Zhengzhou University, He'nan Key Laboratory of Tumor Pathology, Zhengzhou 450052, China; 9 Cancer Care Centre, St George Hospital, Kogarah, and St George and Sutherland Clinical School, University of New South Wales, Kensington, NSW, Australia; 10 Laboratory of Tumor Molecular and Cellular Biology, College of Life Sciences, Shaanxi Normal University, 620 West Chang'an Avenue, Xi'an, Shaanxi 710119, China; 11 CAS Key Laboratory of Nano-Bio Interface, Suzhou Institute of Nano-Tech and Nano-Bionics, Chinese Academy of Sciences, Suzhou 215123, China; 12 Clinical School, Luohe Medical College, 148, Daxue Road, Luohe City, Henan Province, 462000, China; 13 Department of General Surgery, Second Affiliated Hospital of Soochow University, 1055 Sanxiang Road, Suzhou, P.R. China, 215004; 14 GenePharma-Deakin Joint Laboratory of Aptamer Medicine, Suzhou 215123, China and Waurn Ponds, Victoria 3216, Australia

**Keywords:** exosomes, aptamers, theranostics, liquid biopsy, targeting

## Abstract

In the past decade, the study of exosomes, nanosized vesicles (50-150 nm) released into the extracellular space via the fusion of multivesicular bodies with the plasma membrane, has burgeoned with impressive achievements in theranostics applications. These nanosized vesicles have emerged as key players in homeostasis and in the pathogenesis of diseases owing to the variety of the cargos they can carry, the nature of the molecules packaged inside the vesicles, and the robust interactions between exosomes and target cells or tissues. Accordingly, the development of exosome-based liquid biopsy techniques for early disease detection and for monitoring disease progression marks a new era of precision medicine in the 21^st^ century. Moreover, exosomes possess intrinsic properties - a nanosized structure and unique "homing effects" - that make them outstanding drug delivery vehicles. In addition, targeted exosome-based drug delivery systems can be further optimized using active targeting ligands such as nucleic acid aptamers. Indeed, the aptamers themselves can function as therapeutic and/or diagnostic tools based on their attributes of unique target-binding and non-immunogenicity. This review aims to provide readers with a current picture of the research on exosomes and aptamers and their applications in cancer theranostics, highlighting recent advances in their transition from the bench to the clinic.

## Exosomes, a new generation of theranostics

**Figure 1:**
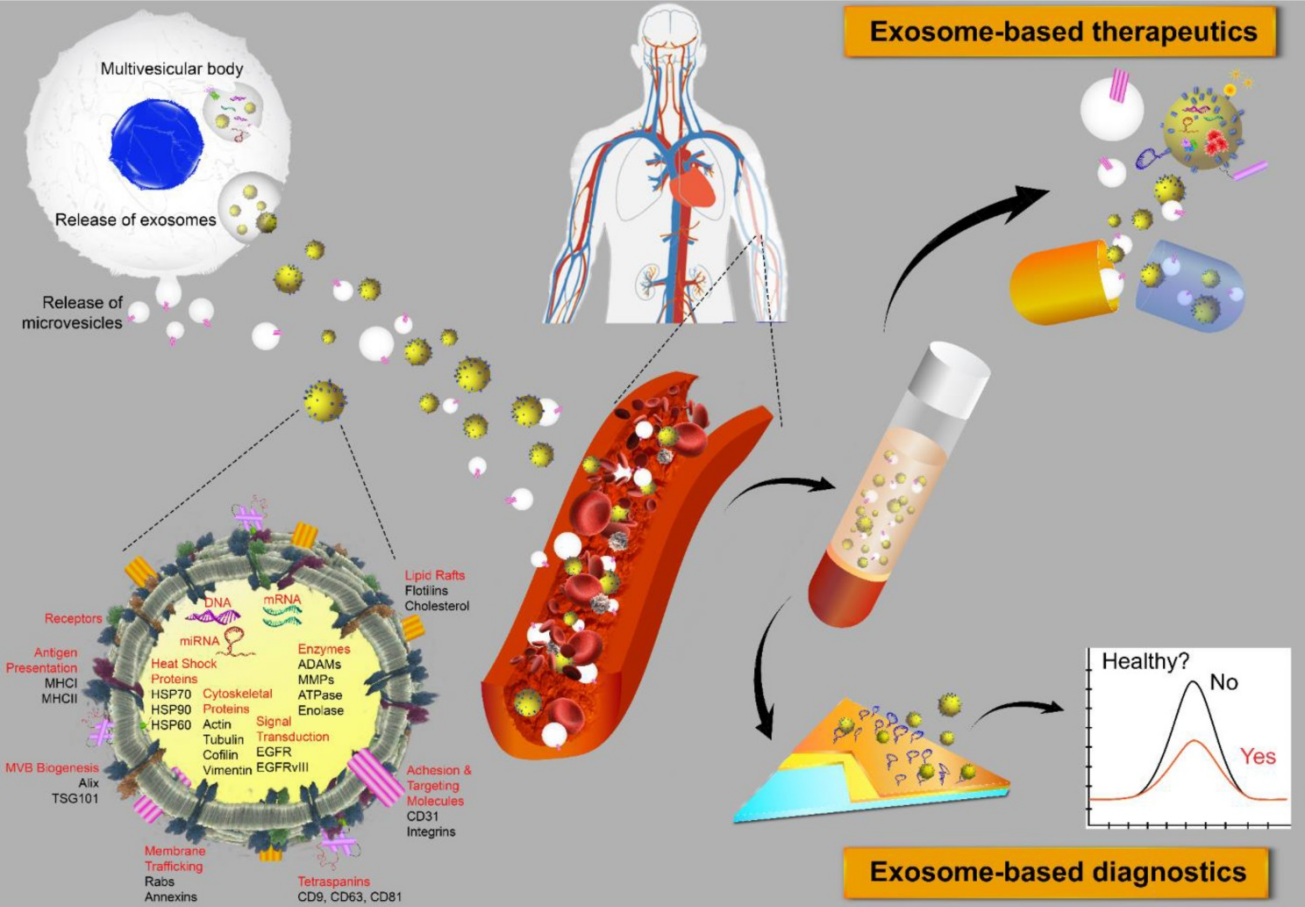
Extracellular vesicles as diagnostic markers and next-generation therapeutics. Exosomes sized 50 to 150 nm are released from most cell types upon fusion of an intermediate endocytic compartment, the multivesicular body, with the plasma membrane. Microvesicles are released by direct budding from the cell surface. Both types of vesicles are composed of an aqueous core and a lipid bilayer membrane and contain a variety of proteins, DNAs, RNAs, lipids and other metabolites. Image of the circulatory system was modified from https://commons.wikimedia.org/wiki/File:Circulatory_System_en.svg by LadyofHats, with permission.

Extracellular vesicles (EVs) are membrane-bound bionanoparticles secreted by all living cells into the extracellular space 1. Based on their mode of biogenesis and size, EVs are classified as exomeres, exosomes, microvesicles, oncosomes or apoptotic bodies 2, 3. From humble beginnings as “platelet-dust” half a century ago 4, EVs have emerged as key mediators for *in vivo* communication among cells, tissues and cross-kingdom molecules 2, 5. Several decades of biochemical and cell biological investigations have culminated in recent works defining exosomes as 50 to 150 nm in size with surface tetraspanins (CD63, CD81, and CD9) as biomarkers and microvesicles as 100 to 1000 nm in size with annexin A1 as a distinct biomarker 6, 7. In fact, exosomes circulating in various cell types have been found in the blood and other body fluids with cargos inherited from the cells of origin. These advances have laid the foundation for exosomes to be a novel source of biomarker discovery. Exosomes are known to carry a number of marker proteins, including heat shock proteins (HSPs), tumor-susceptibility gene 101 (Tsg101), the endosomal sorting complex required for transport (ESCRT-3) binding protein Alix, and major histocompatibility complex (MHC) class I and MHC class II complexes 8, 9. Notably, integrins and other adhesion molecules on the surface of exosomes, such as intercellular adhesion molecule-1 (ICAM-1, also known as CD54) and lymphocyte function-associated antigen 1 (LFA-1) integrin, as well as the exosomal lipid content may facilitate exosome adhesion and fusion with the plasma membrane of recipient cells 10, 11. In addition, the enrichment of specific transmembrane proteins, such as epidermal growth factor receptors (EGFRs) and epithelial cell adhesion molecule (EpCAM), in exosomes reflects their cellular origin 12, 13. These proteins are associated with the normal physiology and pathogenesis of many diseases, leading to their utilization as valuable biomarkers 14. The membranes of exosomes are highly enriched with lipid rafts, which render exosomes highly stable under various *in vivo* and *in vitro* conditions 15. Given the unique lipid composition of their membrane, when compared with that of the cells from which they were derived, exosomes can effectively protect their cargos, such as proteins, mRNA, miRNA, long-noncoding RNA and small nuclear RNA. In addition to their pivotal roles in normal physiology and the pathogenesis of many diseases, exosomes are now poised to become promising next-generation diagnostic and therapeutic tools 16 (**Fig. 1**).

Indeed, exosomes carry information on not only their cells of origin, thus providing readily accessible diagnostic markers, but also the progression and prognosis of a particular disorder. For instance, cancer cell-derived exosomes can carry membrane proteins involved in cancer progression. A recent study on programmed death-ligand 1 (PD-L1) in exosomes from metastatic melanoma cells found that exosomal PD-L1 could inhibit CD8 T cells and facilitate tumor growth 17. Another recent study demonstrated that integrins such as α6, αv and β1, found on cancer cell-derived exosomes could be used to distinguish between different types of cancer, such as breast, kidney, colon and ovarian cancers, and to predict tumor stage, as higher levels of these proteins on exosomes were secreted from the more aggressive progenitor cancer cells 18. Moreover, miRNAs extracted from exosomes can be used as signatures for disease detection and as indicators of the tissue of origin not only in cancer 19 but also in other diseases such as neurodegenerative diseases 20, obesity 21, diabetes 22, lupus nephritis 23, cardiovascular diseases 24, and lung diseases 25. It has been shown that at least some exosomes are able to cross the blood-brain barrier. Thus, the content and number of exosomes in the cerebrospinal fluid and plasma may provide valuable information on the pathogenesis and progression of neurodegenerative diseases 26. In a recent study, plasma exosomes from subjects with HIV-associated neurological disorders and from healthy subjects were isolated using antibodies against the neuronal cell adhesion molecule L1 (L1CAM). The concentration of L1CAM^+^ neuronal exosomes was found to be lower in the neurocognitively impaired participants than that in the subjects without neurological impairment. In addition, increased levels of the neuronal markers neurofilament light (NfL) and synaptophysin were found in the neuron-derived exosomes in plasma from HIV-infected individuals 27. β-amyloid (Aβ) plaques have been regarded as among the earliest hallmarks of the disease, and exosomal β-amyloid in the bloodstream has been utilized for the detection of Alzheimer's disease 28. In addition, CD63/CD66b and CD63/MUC-1 double-positive exosomes in colonic luminal fluid aspirates from patients with inflammatory bowel disease were found to be of neutrophil and epithelial cell origin, respectively, and thus have been utilized as potential fecal biomarkers for mucosal bowel inflammation 29. Interestingly, this dual positive exosome-based approach has also been applied in the development of novel methods for assessing placenta health via the analysis of circulating syncytiotrophoblast exosomes released from the placenta surface that directly entered maternal circulation 30. Exosomes in the circulation that were positive for both a placenta-specific marker (PLAP) and an exosome marker, CD63, were found to gradually increase after 6 weeks gestation, indicating that the release of syncytiotrophoblast exosomes into maternal circulation occurs before the onset of blood flow into the intervillous space, which occurs at approximately 10 weeks gestation in a normal pregnancy 30-32. Furthermore, exosomes harboring proteins involved in innate immunity in saliva and tears were also explored as potential biomarkers of primary Sjögren's syndrome 33, demonstrating the versatile use of exosomes in biofluids as biomarkers for staging and monitoring diseases.

The biological features of exosomes endow these vesicles with the ability to efficaciously deliver therapeutic molecules. The “homing effect” of exosomes indicates that exosomal therapeutics delivered are likely to induce more pronounced anticancer effects in cancer cells from which the exosomes are derived 34. There is also evidence suggesting that mesenchymal stromal cell (MSC)-derived exosomes constitute a promising new modality for the treatment of stroke and traumatic brain injury. In this context, exosomes loaded with selected miRNAs can alter the biological functions of recipient cells 35. Some clinical trials involving exosomes were recently registered, mostly in the contexts of stroke, diabetes, cutaneous wounds and cancer 35. Undoubtedly, we will witness an increasing number of exosome-based therapies in the next few years once the biological functions, molecular mechanism(s) and the safety of exosomes, as well as their manufacturing and associated quality control procedures, are clarified and developed thoroughly.

## Exosome-based liquid biopsy in precision oncology

The survival and quality of life of patients with cancer can be significantly improved when the disease is diagnosed at an early stage. Thus, the early detection of cancer is pivotal for improving treatment outcomes and reducing the cancer burden. The methodologies used for early cancer detection should be sensitive, accurate, minimally invasive and able to be repeatedly performed on demand. To mitigate the limitations of traditional tissue biopsy, a liquid biopsy of biological fluids, such as blood or urine, has been utilized to detect cancer biomarkers with minimal invasiveness, making this technique suitable for obtaining multiple samples over time 36-38. The successful clinical translation of liquid biopsies is exemplified by CellSearch^®^, a diagnostic test for cancer metastasis that detects circulating cancer cells in the blood, which was approved by U.S. Food and Drug Administration (FDA) in 2004. Liquid biopsy is also indispensable for monitoring therapeutic effectiveness in cancer patients undergoing treatment, as well as for providing real-time feedback and guidance on adjustments to optimize treatment regimens. The presence of EVs released by cancer cells in bodily fluids makes them accessible for simple and repeated sampling. Moreover, in some cases, cancer cells release more exosomes into plasma than do healthy cells, and a number of cancer-related biomarkers have been shown to be overexpressed in these cancer-derived exosomes 39, 40. Exosome-associated membrane proteins are valuable for the isolation of exosomes and the characterization of their biological functions in cancer progression 37, 41. The importance of developing potentially novel exosome-based cancer biomarkers has been extensively exploited. The amounts of specific proteins in exosomes, such as glypican 1 42, 43, may constitute biochemical signatures for determining the stage of cancer. Indeed, elevated glypican-1 in circulating exosomes can be used for the early diagnosis of pancreatic cancer as the amount of exosomal glypican-1 correlates with tumor burden 42, 43. In addition, the TYRP2 levels in exosomes were found to be significantly increased in the plasma of patients with stage III melanoma who eventually developed metastases 44. Moreover, L1CAM, CD24 and extracellular matrix metalloproteinase inducers were shown to be increased in exosomes released from ovarian cancer cells in both malignant ascites and serum 45.

One interesting target protein for liquid biopsy development is EpCAM 46. Elevated EpCAM levels are associated with increased cell proliferation, tumor development and progression, as well as with reduced overall survival of cancer patients 47. As an epithelial marker, EpCAM is expressed at low levels in all epithelial cells. The blood of healthy subjects contains very few EpCAM-positive exosomes or EVs 48-50. However, when normal epithelial cells transform into carcinoma cells, significantly more EpCAM-positive EVs are detected in both the general circulation and in other body fluids, such as pleural effusions 51 in patients with carcinomas 52. Hence, circulating EpCAM-positive exosomes could be a minimally invasive diagnostic marker for the early detection of cancers of epithelial origin or carcinomas. Indeed, EpCAM-positive exosomes were harvested from the serum and/or the plasma of lung, ovarian and colorectal cancer patients using an anti-EpCAM antibody to establish novel cancer diagnostic methods 53-55. While EpCAM-positive exosomes can be detected in both patients with benign ovarian disease and ovarian cancer, the exosomal miRNA profiles were remarkably distinct in cancer patients and patients with benign disease 53. Interestingly, the miRNAs found in the exosomes from ovarian cancer patients were abundant and similar among patients, but they were not be detected in healthy subjects 53. Similar results were found regarding the profiles of lung cancer patients and normal controls 54 and of colorectal cancer patients and healthy individuals 55. Of note, miRNAs encapsulated by exosomes are remarkably stable in circulation because exosomes can protect miRNA against RNase-mediated degradation 55. miRNA profiling has emerged as a robust and promising strategy for exosome-based diagnostics, as PCR-based miRNA profiling requires a much lower concentration of exosome than is required for protein-based exosome analysis 55. Indeed, several research groups demonstrated that the association of miRNAs with circulating cancer- derived exosomes could be used as a signature for cancer diagnosis. For example, analyses of exosomal miRNAs revealed a differential expression pattern in patients with prostate cancer compared to controls. Specifically, in the prostate cancer samples, the levels of miR-100-5p and miR-21-5p were found to be valuable for evaluating prostate cancer progression and metastasis 56. Furthermore, tumor-derived exosomal miRNAs, including the adenocarcinoma- specific miR-181-5p, miR-30a-3p, miR-30e-3p and miR-361-5p and the squamous cell carcinoma-specific miR-10b-5p, miR-15b-5p and miR-320b, have been shown to be potential biomarkers useful for distinguishing adenocarcinoma from squamous cell carcinoma in the early diagnosis of non-small cell lung cancer 57. Most recently, a research group from Spain analyzed exosomal miRNA levels in serum samples from 53 women with breast cancer who had been initially diagnosed with localized breast cancer and who were receiving neoadjuvant chemotherapy, before and during the therapy, and compared the levels to those in eight healthy controls 58. Exosomal miRNA-21 and miRNA-21-105 were found to be increased in the metastatic patients compared to the levels in the non-metastatic patients and the healthy donors before receiving neoadjuvant therapy. Importantly, the levels of miRNA-21 were found to be directly correlated with the tumor size during neoadjuvant therapy 58. These intensive studies have culminated in a milestone in exosome-based cancer diagnostics: the FDA approval of the ExoDx^TM^ Prostate IntelliScore (EPI) test in June 2019. ExoDx^TM^, which is an exosomal RNA-based clinical liquid biopsy test based on a simple urine sample that provides a more precise diagnosis of prostate cancer 59-61.

**Figure 2:**
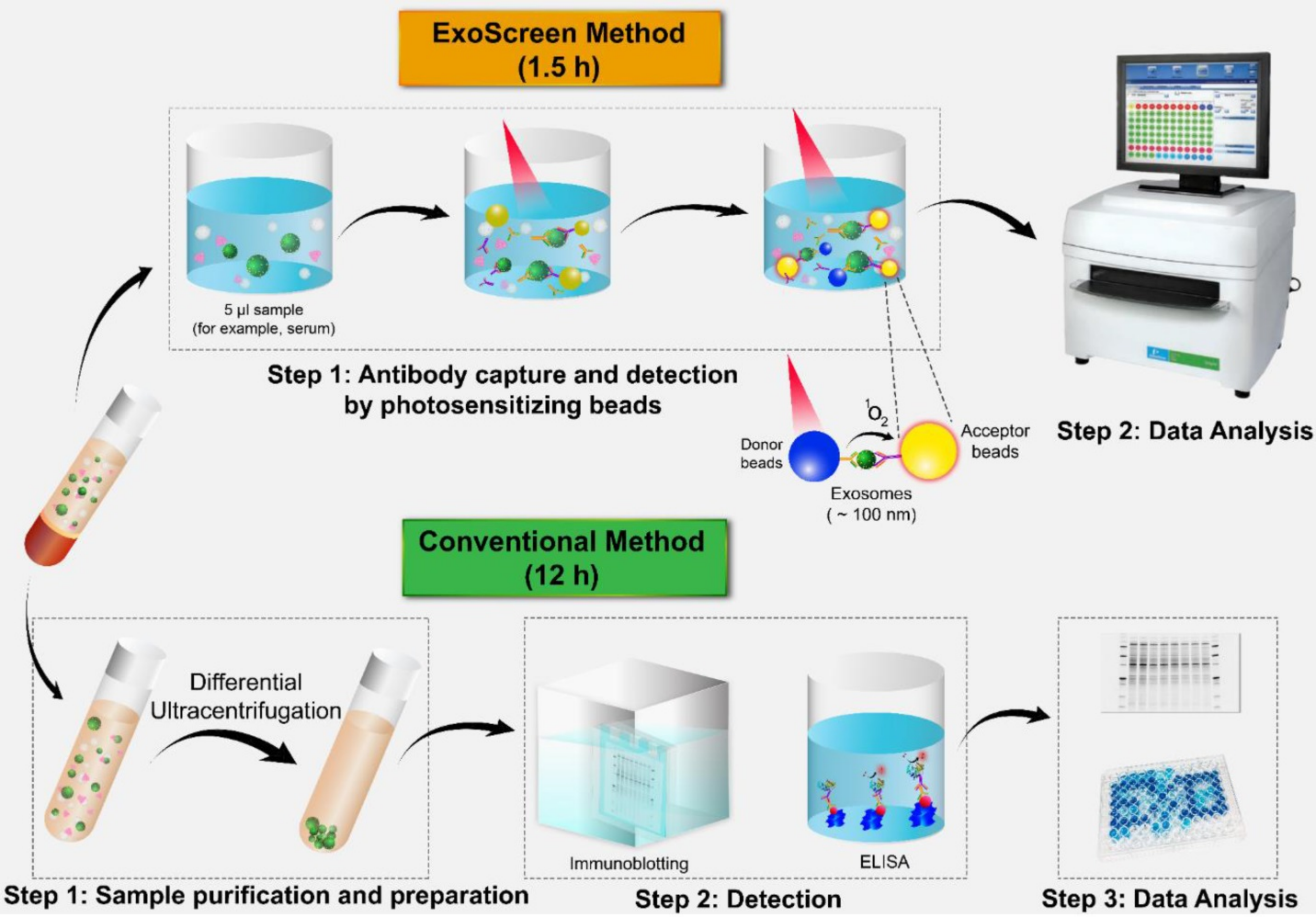
Comparison of ExoScreen and the conventional method of biomarker detection. ExoScreen can be performed without a prior purification step. This system uses photosensitizer beads composed of streptavidin-coated donor beads to capture biotinylated antibodies in the analyte, and these beads are excited with a laser at 680 nm. Singlet oxygen is released and excites the acceptor beads, which are conjugated to a second antibody recognizing an epitope in the analyte, to amplify the fluorescence signal. This system emits light at 615 nm only when (i) exosomes are captured by both antibodies (CD147 and CD9) and (ii) the exosomes are smaller than 200 nm. Images of the plate reader and immunoblotting technique were modified from https://www.perkinelmer.com/category/microplate-readers, and https://www.bioradiations.com/the-how-and-why-of-normalizing-your-western-blots/, respectively, with permissions.

In the past few years, several techniques have been established for the detection of a variety of proteins in exosomes and tumor-associated markers through liquid biopsy. ExoTEST is an enzyme-linked immunosorbent assay (ELISA)-based technique that captures and quantifies exosomes in plasma and other biological fluids by detecting CD63 and Rab5b proteins on exosomes and caveolin-1, a tumor-associated marker 62. In a clinical study of ExoTEST, high levels of exosomes expressing CD63 and caveolin-1 were detected in the plasma of melanoma patients compared with that in the plasma of healthy controls 39. Although exosomes released from cancer cells are thought to promote tumor growth and metastasis, their low abundance in the circulation in the early stages of cancer development constitutes a formidable challenge to the use of liquid biopsy. Nevertheless, employing multiple exosomal biomarkers, Yoshioka and colleagues employed multiple exosomal biomarkers to develop an antibody-based ExoScreen technique (**Fig. 2**) that can be used to monitor CD147-CD9 double-positive exosomes secreted from colorectal cancer cells in only 5 μL of patient serum 63. Interestingly, this method does not require a prior purification step. Thus, ExoScreen is a highly sensitive and rapid liquid biopsy technique for the detection of disease-specific exosomes in circulation.

The inherent limitations of conventional exosome detection methods include the prolonged time, requirement for large amounts of exosomes and extensive post-labeling processes 64. To address these issues, high-throughput approaches have been developed that use nanoplasmonic sensors for label-free detection, molecular profiling and multiplexed phenotyping of exosomal proteins 13, 65. Label-free detection is a portable process that enables point-of-care analyses. For example, a nanoplasmonic exosome (nPLEX) assay was developed based on transmission surface plasmon resonance (SPR) and arrays of periodic nanoholes functionalized with antibodies to identify exosomal CD24 and EpCAM expression in ascites fluid from ovarian cancer patients 13. In addition, a high-throughput-based molecular profiling technique for use with exosomes was established in the format of an exosome array, in which exosomes were first immobilized using antibodies against CD9/CD81/CD63 imprinted on coated glass slides. Subsequently, a panel of antibodies against 21 different cell surface antigens and cancer antigens was added. This method has been successfully applied for exosome profiling using 1-10 µL of plasma from healthy controls and patients with non-small cell lung carcinoma 65, 66. Most recently, Zhang et al. enhanced the efficiency and speed of exosome capture using an ultrasensitive biosensor based on a self-assembled 3D herringbone nanoporous structure; the greater surface area of this sensor increased the number of exosomes that could contact the antibody-coated sensing surface 67. This microfluidic chip was used for the rapid detection of exosomes expressing CD24, EpCAM and folate receptor α (FRα) in only 2 μL of plasma from ovarian cancer patients and healthy controls. This experiment revealed that FRα is a potential biomarker for the early detection and monitoring of ovarian cancer. In addition, the negative charge of exosomes was leveraged to promote electrostatic interactions with nickel at pH values above 5 under physiological conditions. Based on this principle, a simple yet robust strategy for the rapid purification of EVs was developed by combining nickel-based isolates with an amplified luminescent proximity homogeneous assay or droplet digital PCR. This strategy further evolved into an EV-based liquid biopsy used to probe tumor heterogeneity 68. This method was applied to detect picomolar concentrations of exosomal prostate-specific membrane antigen (PSMA) in the plasma of prostate cancer patients. Impressively, the somatic BRAF and KRAS mutations in exosomes circulating in the plasma of metastatic colorectal cancer patients detected by this technique matched the tissue diagnostics with 100% concordance 68. Another new exosomal profiling platform developed by Zhang and coworkers is the ExoProfile chip. This device was constructed of 3D porous serpentine nanostructures via patterned colloidal self-assembly to increase the number of reaction sites and improve the exosome biosensing efficiency. Hence, this device detects a panel of surface protein markers on exosomes, including EGFR, human epidermal growth factor receptor 2 (HER2), CA125, FRα, CD24, EpCAM, CD9 and CD63, with high sensitivity in a multiplexed manner 69. This platform was applied to detect circulating EVs in only 10 μL of plasma from ovarian cancer patients in 3 hours, and the results demonstrated improved accuracy in differentiating early stage from late stage cancer 69. For early cancer detection, Ramshani and colleagues developed a novel platform for the quantification of both free-floating miRNAs and EV-bound miRNAs in plasma, in which a surface acoustic wave lyses exosomes on a microfluidic chip integrated with another concentration-sensing chip that uses an electrokinetic membrane sensor to measure non-equilibrium ionic currents 70. This microfluidic chip is a convenient tool for clinical analysis because it requires no exosome extraction, RNA purification, reverse transcription, or amplification and instead requires only a small volume of plasma (only 20 μL of plasma is needed for exosomal miRNA analysis) and can be performed in approximately 30 minutes with 1 pM sensitivity 70.

While blood is commonly obtained in liquid biopsy, clinicians may request urinary samples for certain types of cancer, such as bladder cancer 71 and prostate cancer 72. Urinary exosomes have been shown to be promising biomarkers of renal-associated pathologies 73. Increased α1-integrin and β1-integrin levels were found in urinary exosomes from metastatic prostate cancer patients compared to those from nonmetastatic patients 74. In addition to its use in combination with tetraspanins (CD63, CD81 and CD9) as a general exosome marker, EpCAM is a specific urine exosomal marker of carcinoma cells that can be captured by antibody-coated magnetic microbeads 75. Campos-Silva and coworkers demonstrated that only 500 μL of urine is required to isolate sufficient exosomes for detecting EpCAM expression by flow cytometry 75. This study described a sensitive and reproducible method for the effective immunocapture of exosomes for future clinical applications. Finally, to address the challenge of the low abundance of urinary miRNAs, Wang and colleagues optimized a number of parameters for droplet digital PCR to achieve much higher sensitivity, reproducibility and accuracy than real-time quantitative PCR 76. The exosome-based liquid biopsy techniques presented in this section are summarized in **Table 1.**

## Superiority of aptamers as targeting ligands

**Figure 3:**
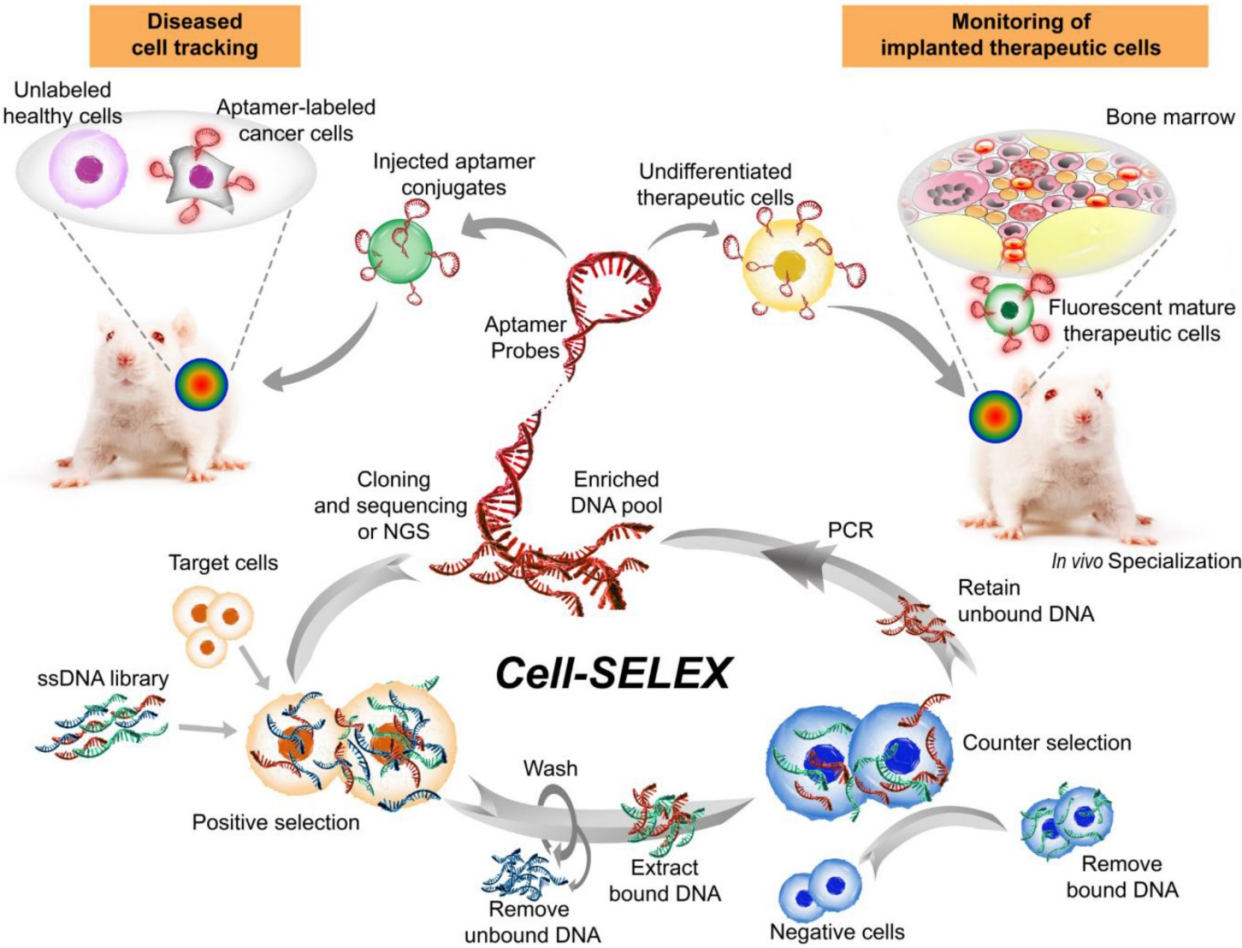
Major steps involved in one cycle of cell-SELEX to isolate aptamer probes for cell tracking. Briefly, after the incubation of a random single-stranded aptamer library with target cells and subsequent washing steps, negative selection is performed to remove nonspecific binding sequences. Subsequently, the target-bound sequences are PCR-amplified prior to being subjected to the next cycle. The aptamers can then be attached to therapeutic cells *ex vivo* to track their biodistribution or differentiation in stem cell therapy following implantation into the host. Alternatively, the labeled aptamers can be used for specifically monitoring target cells. Images of the bone marrow and mice were modified from https://commons.wikimedia.org/wiki/File:Gray72.png by Henry Vandyke Carter and from https://www.stockvault.net/photo/126860/white-mouse by 2happy, respectively, with permissions.

Aptamers, also known as chemical antibodies, are single-stranded DNA or RNA that fold into 3D structures and specifically bind to their targets with high affinity and specificity 80. Aptamers are generated via a PCR-based *in vitro* selection strategy known as systematic evolution of ligands by exponential enrichment (SELEX) 81, 82 (**Fig. 3).** Owing to their advantages over conventional antibodies in terms of low immunogenicity and batch-to-batch variation, high specificity and sensitivity, as well as deeper tumor penetration 83, 84, aptamers have become an invaluable class of affinity ligands in biomedical research, diagnostics 85 and therapeutics 86. For example, a DNA aptamer was developed by Wang and colleagues with superb targeting properties and distinctive functional versatility for early disease detection, imaging, and targeted delivery of therapeutic agents 87. Liu et al. developed a cell-specific DNA aptamer-based fluorescence probe for the molecular subtyping of breast cancer 88. This aptamer probe can distinguish, within 30 minutes, not only between different breast cancer cells but also between breast cancer cells and normal mammary epithelial cells, and between different tumors in mouse xenograft models of human breast cancer 88. Such a robust aptamer has great potential to be further developed into a rapid and sensitive tool for guiding personalized therapy and determining prognosis 89, 90.

Aptamers are emerging as a new class of ligands for tracking tumors at the cellular, subcellular and molecular levels. Aptamers used in super-resolution microscopy offer many advantages over conventional antibody-based immunostaining methods, particularly in cases in which the affinity ligands themselves are larger than the protein of interest. Specifically, aptamer-based probes have improved detection sensitivity due to minimized steric hindrance, increased ligand density and high penetration into cells/tissues 91. Indeed, studies from independent laboratories confirmed that the use of aptamers as detection ligands resulted in superior resolutions well below the light diffraction limit compared to the use of antibodies as probes 91-93. Moreover, to achieve better resolution of aptamer-based probes, Spiegelmer technology has been used to select stable aptamer-based probes that achieve the best imaging quality. In this strategy, endonuclease-sensitive RNA aptamers (D-form) are replaced with endonuclease-resistant RNA aptamers (L-form) or mirror-image aptamers 91, 94.

The *in vivo* targeting of aptamers to tumor cells can be visualized by labeling with a suitable fluorescent agent or radionuclide. ^18^F-radiolabeled HER2-targeted DNA aptamers were developed and demonstrated high tumor uptake. Therefore, such aptamers hold promise as specific HER2-positive tumor imaging agents in positron emission tomography (PET) 95, 96. In addition, FAM and Cy5 were used to label the R13 aptamer to investigate the mechanism of aptamer internalization and the ability to target ovarian cancer cells 97. A bright, orange fluorescent turn-on probe (TMR-DN) bound to a rainbow aptamer, SRB-2, was used to improve the signal-to-background ratio in fluorescence imaging, providing low background fluorescence and enabling no-wash live-cell RNA imaging 98. This novel aptamer-based system was used to image the distinct subcellular localization patterns of ribosomal RNA and mRNA in bacteria and mammalian cells 98. A recent study showed that Cy5-labeled M17, a DNA aptamer that specifically recognizes MMP14, is a promising molecular probe for imaging numerous types of MMP14-positive cancer cells 99. Moreover, the past 10 years have witnessed the development of quantum dot (QD)-labeled aptamers and their extensive applications in cancer theranostics 100, 101. For example, a versatile, sensitive and selective sandwich assay involving a DNA aptamer and QDs as signal amplifiers was established for cancer detection 102. Additionally, EGFR aptamer-conjugated lipid nanoparticles containing QDs and siRNAs were used to achieve remarkable EGFR-dependent siRNA delivery and fluorescence imaging 103.

Simple yet powerful aptamer sensors can be developed on a DNA module platform composed of an aptamer, a joint module for sensing a conformational change in the aptamer, a terminal stem, and a DNAzyme to report target detection in a concentration-dependent manner. Using such a strategy, Tomita and co-workers customized a microarray containing more than 10,000 sequences designed by *in silico* secondary structure predictions for array-based screening 104. Aptamer-based biosensors have demonstrated practical application in point-of-care drug monitoring systems, whereby the circulating drug concentration can be detected in human serum 105. For the design of such a point-of-care device, the biointerface of the sensor may consist of a binary self-assembled monolayer of a specific thiolated aptamer and 6-mercapto-1-hexanol (MCH, a surface blocking agent and aptamer spacer) at an optimized ratio based on electrochemical impedance spectroscopy measurements, which are used to enhance the sensitivity towards specific targets 106. Memristive nanowires, assembled based on the regeneration properties of DNA aptamers on their surface, have also emerged as potential real-time monitoring tools for ultrasensitive, highly specific and selective drug biosensors. Such systems have been used for the successful detection of tenofovir in human serum 107. Electrochemical aptamer-based sensors constitute another type of promising biosensor for the rapid, specific recognition and quantification of drugs such as insulin. In this device, a redox label-modified guanine-rich aptamer that folds into a G-quadruplex serves as as a probe to detect insulin, enabling researchers to discriminate insulin, glucagon and somatostatin in a Krebs-Ringer bicarbonate buffer 108. Another sandwich-type electrochemical aptamer-based biosensor, known as an aptasensor, was designed with a combination of tetrahedral DNA aptamers and a flower-like nanozyme/horseradish peroxidase (HRP) combination and used for the detection of the breast cancer cell biomarker HER2 109. Specifically, in the assembly of this biosensor, the aptamer specifically binds to HER2 and the complex is immobilized on the gold electrode surface, where the Mn_3_O_4_- Pd@Pt nanozymes are linked by another aptamer and natural enzyme HRP, collectively referred to as nanoprobe 1, to amplify the biosensor signal. To further amplify the signal, nanoprobe 1 is subsequently converted into dendritic DNA nanostructures via links with nanoprobe 2, a structure based on Pd@Pt/HRP/cDNA 109.

Since the development of aptamer technology nearly 30 years ago, pegaptanib, an RNA aptamer against vascular endothelial growth factor (VEGF), is the only aptamer that has been approved by the FDA for the treatment of macular degeneration 110. Nevertheless, a number of aptamers are undergoing preclinical and clinical development, including aptamers to thrombin, nucleolin and prostate-specific membrane antigen 111. Three strategies have been commonly used for the therapeutic application of aptamers, namely, (i) aptamers as antagonists, (ii) aptamers as agonists, and (iii) aptamers as delivery agents 112. All the aptamers in current clinical trials have been classified as category (i) aptamers, and the design of most must be improved to meet the expectations for therapeutic efficacy, such as the generation of a hybrid complex with an antibody 113, conjugation with cholesterol 114 or nanoparticles 115, or formulation as a multimer 116. On the other hand, a few aptamers have been developed as agonists, and they include RNA aptamers against HER3/ERBB3, OX40 (CD134), 4-1BB (CD137), CD40 and CD28 and DNA aptamers targeting human VEGFR-2 and insulin receptor 112. As targeting ligands in drug delivery systems, aptamers function to guide the systems to the desired cells/tissues to maximize treatment efficacy and minimize systemic toxicity 117. By neutralizing histones with chemically stabilized anionic 2′ fluoro-modified RNA aptamers with high binding affinity, Giangrande's group demonstrated the efficacy of histone-specific aptamers in the treatment of multiple clinical conditions associated with multiple organ dysfunction syndrome 117. One new tool generated by aptamer-based techniques for nanomedicine precision therapy is a DNA nanorobot. Ma et al. generated a new intelligent DNA nanorobot based on an anti-HER2 aptamer anchored to a tetrahedral nucleic acid framework for the selective lysosomal degradation of HER2 protein in breast cancer cells 118.

Although aptamers are promising ligands for targeted theranostics, one potential barrier for their translation into the clinic is the possible loss of targeting capacity under *in vivo* conditions. After performing studies to mitigate this concern, Tan's group recently reported the causes of this loss of targeting 119. By determining variations in the surface chemistry and biological behavior of nanoparticles in serum, Tan's group identified several factors that contribute to the loss of efficacy of aptamer-guided nanocarriers. The rapid clearance of nanoparticles due to the immune response to the nanoparticle surface, aggregation of small nanoparticles, protein corona blocking, and enzymatic cleavage of the aptamer 119 are among the key mechanisms underlying the loss of aptamer-based targeting efficacy *in vivo*. Future smart design and engineering are expected to provide effective solutions to this problem.

## Aptamer-guided exosome diagnostics and therapeutics

The application of aptamer technology in both basic science research and the clinical translation of exosomes is poised to lead to the development of the next generation of diagnostics and therapeutics.

### Aptamer-guided exosome diagnostics

To exploit molecular markers on the surface of exosomes for improving exosome enrichment efficiency and facilitating exosome isolation, aptamers can be selected to specifically bind to the markers and thus capture the exosomes. The engineering of aptamer-guided exosome diagnostic tools is outlined in the following sections.

#### Simple use of peptide/RNA/DNA aptamers to capture exosomes

By selecting an aptamer against an overexpressed protein on exosomes, an aptamer-guided exosome-capturing nanoplatform system can be developed as a diagnostic tool. Garrido and colleagues developed an aptamer against the A8 peptide that could bind to the extracellular domain of HSP70, which is overexpressed in many cancers 120. This aptamer was utilized to capture HSP70-positive exosomes in urine samples from patients with breast, lung, or ovarian cancer 121. This study demonstrates a key advantage of quantifying cancer-derived exosomes over determining the number of circulating tumor cells: there are more exosomes than cancer cells in systemic circulation. Moreover, exosomes can be quantified in both blood and urine. Murakami et al. selected two RNA aptamers of 55 and 30 nucleotides (nt) after several rounds of SELEX 122. Both the 55- and 30-nt aptamers had strong affinity for exosomes, as analyzed by SPR, whereas circular dichroism spectroscopy revealed that the two aptamers could form a G-quadruplex structure in their loop regions that was stabilized by potassium ions 122. In a separate work, Sun's group developed λ-DNA- and DNA aptamer-mediated approaches for the simultaneous size-selective separation and surface protein analysis of exosomes 123. Impressively, a machine learning algorithm applied to exosomal size and marker signatures was able to distinguish various breast cell lines and stage II breast cancer patients with varied HER2 expression patterns 123.

#### Aptasensor

**Figure 4:**
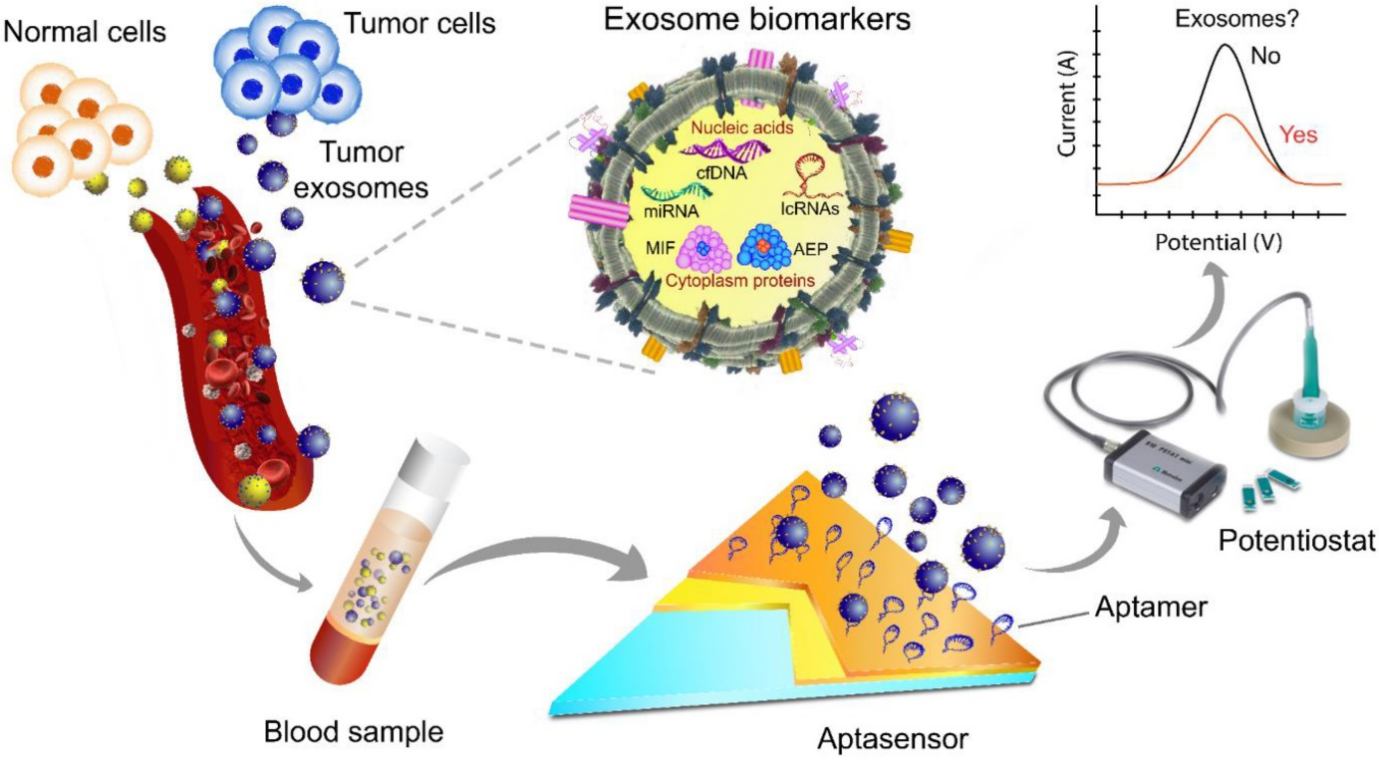
A scheme of exosome isolation and/or analysis via an electrochemical aptasensor. In this platform, the electrode is made of gold and carbon surfaces for the detection of different target analytes, e.g., tumor-derived exosomes of various sizes. Changes in the redox signal are proportional to the concentration of exosomes. After aptamer immobilization followed by incubation with exosomes, the signal is typically significantly suppressed due to the decreased electrode surface area. The image of the potentiostat was modified from https://mep.metrohm.com.au/2017/07/29/metrohm-autolab-portable-potentiostat-kit/ with permission.

Given their advantages of high sensitivity, rapid response, portability, and low sample volume requirement, aptasensors have become an important diagnostic tool for detecting cancer-derived exosomes 124 (**Fig. 4**).

Wang et al. presented a nanotetrahedron (NTH)-assisted electrochemical aptasensor as a sensitive and rapid tool for the direct capture and detection of exosomes secreted by hepatocellular carcinoma cells 125. In this study, an aptamer containing one expanded nucleotide was generated against liver cancer cells, and it exhibited selectivity for exosomes secreted from HepG2 cells. The NTH-assisted aptasensors had improved specificity and capture efficiency. This study provides a strategy for overcoming the limitations of aptasensors that impede exosome detection, including the likelihood that immobilized aptamers on electrodes undergo self-assembled monolayer aggregation or entanglement and the inability to control the spatial orientation of single-stranded aptamers 125. Another technical challenge relates to the efficient detection of cancer-derived exosomes among a background of exosomes secreted by non-cancerous cells in order to evaluate the diagnostic results or to gain a comprehensive understanding of the tumor migration and/or invasion mechanism. To overcome these challenges, Huang et al. developed a label‐free electrochemical aptasensor containing an anti‐CD63 antibody-modified gold electrode and an aptamer against mucin. The mucin aptamer was linked to a primer sequence complementary to a G‐quadruplex circular template and was used to detect gastric cancer exosomes 126. The high sensitivity of this method was based on the effects of the hemin/G-quadruplex DNAzyme on H_2_O_2_ reduction to influence the production of electrochemical signals as well as a signal amplification step. This is achieved through rolling circle amplification, a reaction that generates high molecular weight products to accomplish signal amplification 126. Such an electrochemical aptasensor has a detection limit of ~ 100 cancer biomarker-positive exosomes per milliliter.

#### Other lab-on-a-chip-based diagnostic tools

Most aptamer-guided exosome diagnostic tools are based on the principle of selective binding between aptamers and exosome/disease markers. Inorganic materials and polymers are common components used in conjunction with aptamers to form integrated platforms for lab-on-a-chip exosomal diagnostic devices. Zhang and colleagues developed the ExoAPP assay, an aptamer nanoprobe-based exosome profiling system that can be used to phenotype exosome surface proteins and quantify cancer-derived exosomes 127. This tool consists of a graphene oxide interfacing with aptamers against exosome markers. The profiling of exosome markers is achieved by the integration of enzyme-assisted exosome recycling. This assay can detect exosomes at the limit of 1.6 × 10^5^ particles/ mL. The ability of ExoAPP to identify surface PSMA on target exosomes in blood samples from prostate cancer patients is indicative of its future application in clinical diagnostics 127. Furthermore, to enrich EpCAM-positive cancer-derived exosomes for clinical detection, Yoshida et al. developed an EpCAM‐affinity coating agent (EpiVeta) to be used on the surface of inorganic materials in diagnostic devices 128. EpiVeta consists of a conjugate of a peptide aptamer for EpCAM and an MPC polymer. The aptamer enables the versatility of the conjugation process, while the zwitterionic membrane‐mimicking polymer MPC consists of methacrylate with a phosphoryl‐choline polar group and is used to reduce the non‐specific binding of proteins to material surfaces 128. In addition, Xu et al. reported a two-stage microfluidic platform termed the ExoPCD chip for EV on-chip isolation and *in situ* electrochemical analysis of exosomes from human serum using DNA aptamer sensors 129. The ExoPCD chip utilized magnetic enrichment based on the specific recognition of the phosphatidylserine-Tim4 protein combined with a novel signal transduction pathway to detect CD63-positive exosomes with high sensitivity using only 30 µL of serum. Samples from liver cancer patients and healthy controls were distinguished by this chip, demonstrating that it is a promising tool for exosome analysis and non-invasive diagnostics 129. Additionally, in developing a diagnostic tool based on the recognition of the surface protein CD63, Wang et al. fabricated a device consisting of three surface- enhanced Raman scattering (SERS) probes coupled with CD63 aptamer-based magnetic substrates for screening and detecting a broad range of exosomes 130. Importantly, to simultaneously detect multiple types of exosomes, the gold nanoparticle probe was modified with different Raman reporters, each with a specific aptamer targeting the exosomes of interest. This system demonstrates great potential for further clinical applications in cancer screening because it can successfully detect target exosomes in blood samples from patients with breast cancer, colorectal cancer or prostate cancer 130.

#### Challenges to be addressed

Numerous new platforms have been developed to address the technical challenges in conventional methodologies regarding separation/isolation/c apture of exosomes. Researchers at the Chinese Academy of Sciences have developed an aptamer- based fluorescence polarization assay with a one-step mix-and-read platform for exosome quantification that requires less than 1 µL of plasma and has no purification or amplification step 131. This technique, named the AFPExo assay, was developed to overcome the problem of exosome loss during conventional purification/isolation steps, such as ultracentrifugation, ultrafiltration and size exclusion chromatography. This assay is based on the principle that the molecular mass of dye-labeled aptamers is significantly altered after exosome binding, causing a significant change in the fluorescence polarization signal as analyzed with a plate reader. Furthermore, such signals are amplified by the inherent large mass/volume of exosomes. This assay can analyze as many as 5 × 10^5^ exosomes in 1 μL within 30 minutes with a detection limit of 500 exosomes per microliter 131.

Furthermore, it is often desirable or necessary to release the captured exosomes from aptamers immobilized on a solid matrix. This can be achieved by the use of chelating agents, such as EDTA, which removes magnesium ions and disrupts the 3D structure of the aptamer, resulting in the release of the bound target. Unfortunately, such a chelating agent-based strategy may not work with all aptamers. Ideally, an affinity-based isolation technique should deliver intact exosomes that retain structural integrity and biological function for further downstream applications. To this end, Zhang and colleagues designed a CD63 aptamer- and magnetic bead-based approach that can be used to rapidly isolate exosomes and subsequently release intact captured exosomes within 90 minutes through the use of a DNA structure-switch aptamer 132. The functional competence of the exosomes released after capture was confirmed with a wound healing assay 132.

### Aptamer-guided exosome therapeutics

**Figure 5:**
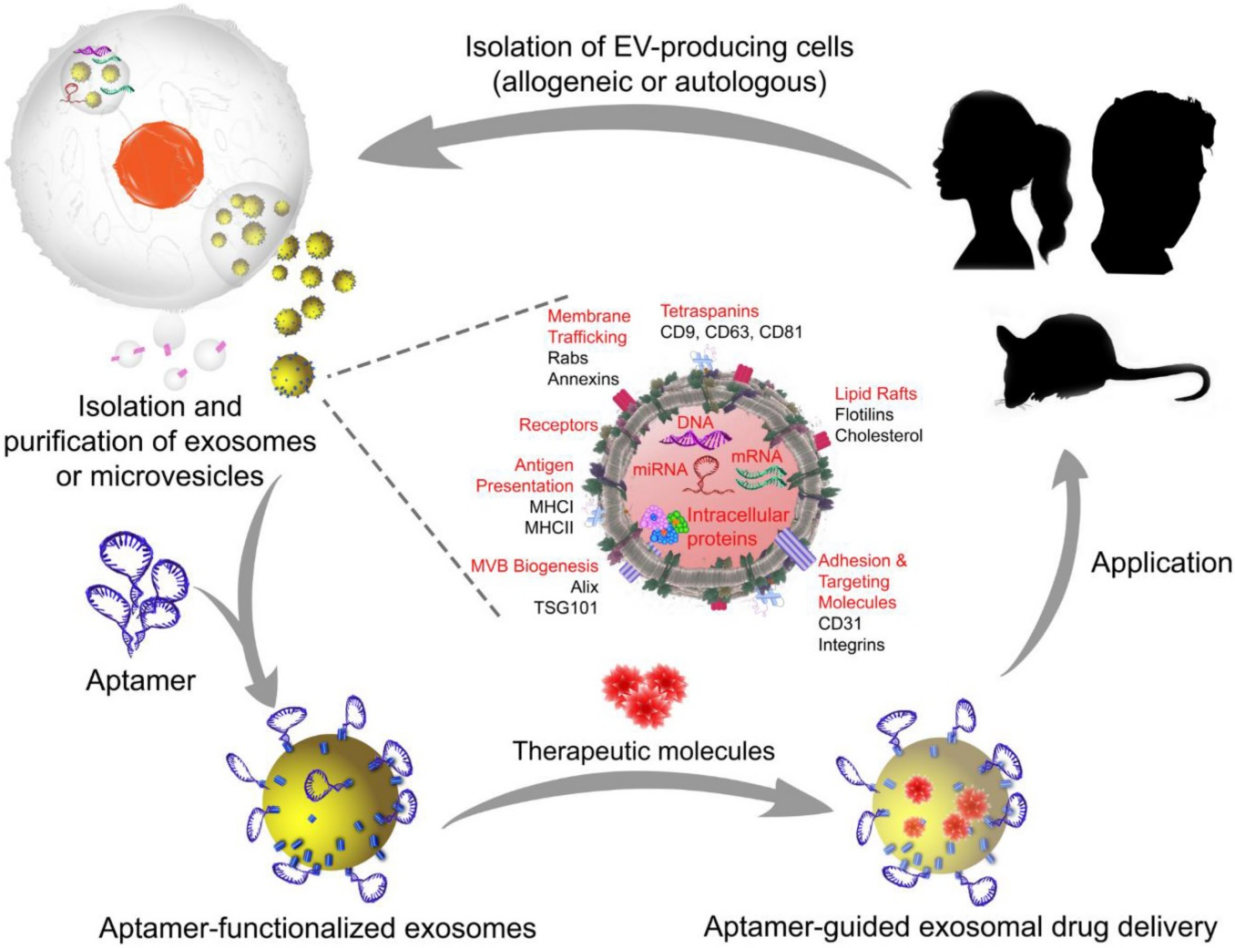
A typical manufacturing cycle of the EV-based engineering of exosomes or microvesicles for targeted therapeutics. In allogeneic EV therapy, EV-producing cells are isolated from individuals who are not genetically identical and then manipulated *in vitro* to load the therapeutic cargo. Subsequently, aptamers are decorated on the exosome or microvesicle surface to generate aptamer-targeted exosomes or microvesicles that are ready to deliver therapeutic cargos in a targeted system. In autologous EV therapy, the EV-producing cells are obtained from the patient requiring treatment, as EVs can be readily isolated from body fluids or produced by cultured mesenchymal stem cells from the patient, and then transferred back to the same patient after *in vitro* cargo loading and surface functionalization with aptamers. Images of the silhouette (mouse and girl) were downloaded for free from https://www.nicepng.com/ourpic/u2q8y3a9a9i1a9y3_mouse-silhouette-sidewasy-mouse-clip-art-silhouette-mouse/, and https://www.freepik.com/free-vector/black-girl-silhouettes_764970.htm#page=1&query=girl%20silhouette&position=1, designed by Freepik.

While the technologies for aptamer-based exosome diagnostics have been developed extensively, relatively few studies that have evaluated aptamer-targeted exosome delivery systems have been reported. Wan et al. reported aptamer-targeted exosomes carrying paclitaxel, an anticancer drug commonly included in chemotherapy regimens, as a new platform for clinical implementation in cancer treatment 133. In this study, the nucleolin-targeting aptamer AS1411 was covalently conjugated to cholesterol-PEG and subsequently grafted onto mouse dendritic cell membranes. These cells were then mechanically extruded to create aptamer-guided exosomes. With this extrusion-based strategy, approximately 3 × 10^10^ aptamer-guided exosomes with a peak size of 105 nm were obtained from ~1 × 10^7^ cells within one hour. Cholesterol-PEG2000 was chosen because of its amphiphilicity and relative rigidity to stabilize exosomes through hydrophobic- hydrophobic interactions at the lipid bilayer. The strategy for synthesizing the cholesterol-PEG2000- aptamers could potentially be utilized for large-scale production of targeted exosomes secreted from immune cells for the treatment of different cancers. This methodology was proposed to be potentially safer than cell-based immunotherapy (e.g., CAR T-cell therapy) because the cells used to prepare the exosomes can be obtained from the patients who need the therapy. Such patient-derived exosomes can be used for drug loading without the need for genetic engineering 133. In an alternative approach, the orientation of the aptamer ligand on the surface of the exosomes was used to reprogram exosomes to control siRNA/miRNA loading and surface ligand display on exosomes with an RNA aptamer, which resulted in efficient targeted exosome delivery and cancer suppression 134. Specifically, these authors engineered an RNA nanostructure with a three-way junction to control the ligand display on the surface of the EVs. The placement of membrane-anchoring cholesterol at the tail of the three-way arrow resulted in the display of the RNA aptamer or folate on the outer surface of the vesicles. In contrast, the placement of cholesterol at the arrowhead of the three-way arrow resulted in partial loading of RNA nanoparticles into the vesicles. As a result, the RNA nanostructure was directionally linked to the lipid bilayer membrane of the EVs, and the targeting ligands decorated the outer surface of the EVs. Such orientation-engineered ligand-displaying EVs could specifically deliver siRNA to cells and achieve an efficient blockade of tumor growth in the three cancer models studied 134. Most recently, the aptamer sgc8, which can specifically recognizes membrane-bound protein tyrosine kinase 7 (PTK7), was conjugated to a diacyllipid via a PEG linker in a targeted anticancer therapeutic delivery platform 135. The exosomes secreted by immature dendritic cells were loaded with doxorubicin by electroporation, followed by the surface functionalization of vesicles with the targeting ligand via the hydrophobic interaction between the diacyllipid tail of the aptamer conjugates and the phospholipid bilayer of the exosomes 135. Such engineered sgc8-guided exosomes showed selective and dose-dependent cytotoxicity to human leukaemia (CEM) cells. Regarding the cellular uptake mechanism, studies showed that among multiple endocytosis pathways, clathrin-mediated endocytosis played a major role in sgc8 aptamer-mediated cellular internalization. These results indicate that the targeting ligands themselves may affect the interaction of exosomes with cells 135. However, whether other aptamer-target pairs also affect exosome uptake by various types of cancer cells remains to be ascertained. Figure 5 illustrates a typical therapeutic application of aptamer-guided EVs *in vivo*.

### Commentary on aptamer-guided exosome theranostics

The capture of exosomes using aptamers against exosomal markers such as tetraspanins and other cancer-specific markers, followed by the quantitative assessment of a disease-specific marker, constitutes an invaluable strategy for detecting disease at an early stage, monitoring the treatment response and making accurate prognostic predictions. The fact that there are more circulating exosomes in the peripheral blood of tumor patients than in that of healthy subjects suggests that tumor-derived exosomes are a potentially useful noninvasive diagnostic tool. The commonly used techniques to detect and quantify individual exosomes include nanoparticle tracking analysis (NTA) and flow cytometry. While the results obtained from NTA are subject to interference from contaminating lipoproteins and protein aggregates, which are similar to exosomes in size and density, most flow cytometers are unable to detect vesicles smaller than 100 nm. On the other hand, the specific detection of exosomal surface proteins enables an effective determination of exosomes. However, ELISA requires a relatively large number of exosomes with a detection limit of approximately 10^7^ particles/ μL. Fortunately, fluorescence and electrochemical platforms with specifically designed aptamers can be highly sensitive. Compared with antibodies, aptamers have superior performance because they are much smaller. For instance, an EpCAM aptamer was shown to have better tumor penetration, more homogeneous distribution and better retention *in vivo* than an EpCAM antibody in a mouse xenograft tumor model 84. Indeed, antibodies have poor access to intracellular targets and are more immunogenic and thermally unstable 136. Other limitations of antibodies have been extensively reported, including the high production costs, unexpected pharmacokinetic profiles, unclear modes of action, poor tissue penetration and heterogeneous distribution 137, 138. In contrast, aptamers have high affinity for the targeted exosomal membrane proteins 139. Moreover, exosomes have a molecular mass of approximately 3.3 × 10^7^ kDa 140, and aptamers are much smaller than antibodies (6~10 kDa vs. 150 kDa) 141. An IgG molecule occupies an area of ~77 nm^2^, while a 25-nt aptamer covers an area of ~ 10 nm^2^. If both the antibody and 25-nt aptamer were conjugated to a single fluorophore or HRP, then the exosome surface area covered by one antibody would be equal to that of multiple aptamers if all other parameters were similar (e.g., equilibrium dissociation constant, on-rate and off-rate). Therefore, the use of aptamers as binding ligands in lieu of using antibodies will allow the more robust isolation and detection of exosomes. In addition, nucleic acid chemistry enables the large-scale and economical synthesis of aptamers with minimum batch-to-batch variations. Due to these distinct advantages, aptamers are becoming a highly attractive class of targeting ligands for engineering exosomes in theranostic applications (summarized in **Table 2**).

## Prospects for next-generation theranostics

Since its humble beginnings 30 years ago, aptamer technology is now paving the way for the flourishing development of new and effective diagnostic and therapeutic tools for major diseases 142-146. Taking advantage of their small size compared to antibodies and their exquisite binding affinity and specificity 112, aptamers can bind to some otherwise inaccessible targets, thereby increasing tissue penetration and conferring enhanced therapeutic efficacy 147, 148. Chemical engineering and nanotechnological efforts have led to the generation of chemically modified aptamers with improved pharmacokinetic profiles *in vivo* 114. Moreover, with their advantages of cost-efficiency, time-saving, minimal batch-to-batch variation and with the ability to release intact exosomes after purification, aptamers have become robust tools for both basic research and clinical translation in exosome-based targeted theranostics 112, 121. Furthermore, aptamers against disease-specific exosomes can be selected using modified SELEX techniques such as counter-SELEX to enhance the specificity 149, thereby empowering targeted exosome therapeutics. At the diagnostic front, a number of advanced diagnostic tools for exosome isolation and/or detection have emerged to provide unprecedented sensitivity and specificity, such as nPLEX 13, the fluorescence-based microfluidic chips 150, the alternating current electrokinetic microarray chip 151, and interferometric imaging 152. Future work will focus on expanding the development of assays for accurate, reproducible, sensitive, and rapid EV detection without a requirement for separation and washing steps. Eventually, clinically validated assays must be adapted for direct implementation in modern automated pathology laboratories. Ideally, some of these next-generation diagnostics will be adapted as hand-held devices for point-of-care use.

A key challenge in exosome research and development is exosome heterogeneity because each exosome is unique in its molecular and biochemical composition 153. Currently, there is an acute lack of robust methods for isolating or purifying exosomes and microvesicles for both diagnostic and therapeutic applications. Although most published work in the field of EV research has focused on exosomes, hence the exosome-centric coverage in this review, microvesicles have emerged as key players in mediating intercellular communication between cancerous and stromal cells and in orchestrating complex pathophysiological processes 154-159. Therefore, the next decade will witness renewed efforts in the development of microvesicle-based theranostics. Indeed, recent seminal work by Coffey and colleagues, for the first time, has established CD81/CD9/CD63 and annexin A1 as specific biomarkers for exosomes and microvesicles, respectively 7. The future development of aptamers against these newly identified EV markers will greatly enhance our ability to effectively and efficiently isolate EV subpopulations. We will then be able to elucidate the mechanisms underlying EV biogenesis and heterogeneity and translate the results of EV research to pathology laboratories and the clinic.

## Figures and Tables

**Table 1:** Typical and most recent techniques of EV-based liquid biopsy with potential applications to cancer diagnosis.

Technique	Biomarker	Cancer	Volume of samples	Time	Limit of detection	Reference
ExoDx^TM^ Prostate (The first exosome-based liquid biopsy test approved by the FDA)	ERG, PCA3, and SPDEF (internal reference)	Prostate	~ 20 mL (urine)	NA	prostate-specific antigen 2-10 ng mL^-1^; SPDEF detected at >30 copies per reaction	61, 77
ExoDx^TM^ Lung (ALK)	EML4-ALK	Lung	0.9-1.5 mL (plasma)	NA	2.5 copies per reaction	78, 79
ExoTEST	CD63, Rab5b, and caveolin-1	Melanoma	100 µL (plasma)	~ 3 days	less than 50 pg of targeted exosomal protein	39
ExoScreen	CD9, CD63, CD147, CEA, and CA19-9	Colorectal	5 µL (serum)	~ 120 min	NA	63
Nanoplasmonic assay (nPLEX)	CD24, CD41, CD45, CD63, CA125, CA19-9, D2-40, EpCAM, EGFR, HER2, CLDN3, and MUC18	Ovarian	NA (ascites)	~ 30 min	~ 3000 exosomes	13
EV array	~ 60 biomarkers, including tetraspanin markers, EpCAM, NY-ESO-1, MUC16, CEA, CD151, CD142, CD146, EGFR, PDL-1, MET, and p53, CD13	Non-small cell lung carcinoma	1-10 µL (plasma)	~ 3 days	2.5 × 10^4^ per microarray spot (~1 nL)	66
Microfluidic chip (self-assembled 3D herringbone nanoporous structure)	CD24, EpCAM, and folate receptor alpha proteins	Ovarian	2 µL (plasma)	~ 90 min	10 exosomes μL^-1^	67
Nickel-based isolation (NBI)-alpha or digital PCR	PSMA, KRAS, and BRAF	Prostate, colorectal	20 µL (plasma)	~180 min	NA	68
ExoProfile chip (3D porous serpentine nanostructures)	EGFR, HER2, CA125, FRα, CD24, EpCAM, CD9, and CD63	Ovarian	10 µL (plasma)	~180 min	21 exosomes μL^-1^	69
Surface acoustic wave (SAW)-based microfluidic chip	miR-21, miR-550, and miR-146a	Liver, pancreatic, oral	20 µL (plasma)	~ 30 min	1 pM (miRNA concentration)	70
Antibody-coated magnetic microbeads followed by flow cytometry	CD63, CD81, CD9, and EpCAM	Prostate	500 µL (urine)	~ 2 days	30 ng of exosomes (1.37 × 10^7^ particles)	75
Droplet digital PCR	miR-29a	Healthy volunteers	0.3-2 µL (urine)	NA	5 copies μL^-1^	76

**Table 2:** Summary of aptamers used in aptamer-guided exosome theranostics.

	Aptamer	Cells producing exosomes (protein target)	Clinical samples/ potential applications	Reference
**Diagnostics**	A8	HCT116, SW480, PC3, HeLa (HSP70)	Urine from patients with breast, lung, or ovarian cancer	121
	MO-1, MO-2	293T, HeLa S3 (tetraspanin)	Cervical cancer	122
	H2 and SYL3C	BT-474 and SK-BR-3 (HER2 and EpCAM)	Breast cancer	123
	LZH8	HepG2	Hepatocarcinoma	125
	MUC_3	SGC7901 (Mucin 1)	Plasma from gastric cancer patients	126
	EpCAM/ Ep114	HT-29, HCT-15, and MCF-7 (EpCAM)	Colorectal, and breast cancer	127, 128
	CD63	HepG2, A549, and MCF-7	Serum from patients with gastric cancer, plasma from patients with non-small lung cancer or breast cancer	127, 129, 131, 132
	H2, CEA, and PSMA	SKBR3 (HER2), T84 (carcinoembryonic antigen), and LNCaP (PSMA)	Blood from patients with breast cancer, colorectal cancer, or prostate cancer	130
**Therapeutics**	AS1411	Dendritic cells (nucleolin)	Breast cancer	133
	PSMA and EGFR	HEK293T	Breast, prostate, and colorectal cancer	134
	sgc8	Dendritic cells (PTK7)	Leukemia/ Lymphoma	135

## Abbreviations

Aβ: β-amyloid
CA125: cancer antigen 125
cDNA: complementary DNA
EGFRs: epidermal growth factor receptors
ELISA: enzyme-linked immunosorbent assay
EpCAM: epithelial cell adhesion molecule
EPI: ExoDx^TM^ Prostate IntelliScore
ESCRT: endosomal sorting complex required for transport
EV: extracellular vesicles
FDA: Food and Drug Administration
FRα: folate receptor α
HER2: human epidermal growth factor receptor 2
HRP: horseradish peroxidase
HSPs: heat shock proteins
ICAM-1: intercellular adhesion molecule-1
L1CAM: neural cell adhesion molecule L1
LFA-1: lymphocyte function-associated antigen 1
MCH: 6-mercapto-1-hexanol
mRNA: messenger RNA
miRNA: microRNA
MHC: major histocompatibility complex
MMP: metalloproteinase
MSC: mesenchymal stromal cell
MUC-1: Mucin-1
NfL: neurofilament light
nPLEX: nanoplasmonic exosome
nt: nucleotide
NTA: nanoparticle tracking analysis
NTH: nanotetrahedron
PCR: polymerase chain reaction
PD-L1: programmed death-ligand 1
PET: positron emission tomography
PLAP: placental alkaline phosphatase
PSMA: prostate-specific membrane antigen
PTK7: protein tyrosine kinase 7
QD: quantum dot
SELEX: systematic evolution of ligands by exponential enrichment
SERS: surface-enhanced Raman scattering
SPR: surface plasmon resonance
siRNA: small interfering RNA
TMR-DN: tetramethylrhodamine-dinitroaniline
Tsg101: tumor- susceptibility gene 101
TYRP2: tyrosinase-related protein-2
VEGF: vascular endothelial growth factor
